# Use of Dried Blood Spots for Identifying Hepatitis E Infections in an Outbreak Setting

**DOI:** 10.1002/jmv.71155

**Published:** 2026-09-15

**Authors:** Catia Alvarez, Robin Nesbitt, Kinya Vincent Asilaza, Pascale Sattonnet‐Roche, Joseph Wamala, Shimul Das, Melat Haile, Monica Rull, Etienne Gignoux, Manuel Albela, John Rumunu, Isabella Eckerle, Iza Ciglenecki, Andrew S. Azman, Benjamin Meyer

**Affiliations:** ^1^ Geneva Centre for Emerging Viral Diseases, Geneva University Hospitals Geneva Switzerland; ^2^ Department of Medicine, Faculty of Medicine University of Geneva Geneva Switzerland; ^3^ Department of Microbiology and Molecular Medicine, Faculty of Medicine University of Geneva Geneva Switzerland; ^4^ Epicentre Paris France; ^5^ Médecins Sans Frontières Juba South Sudan; ^6^ World Health Organisation Juba South Sudan; ^7^ Médecins Sans Frontières Geneva Switzerland; ^8^ Ministry of Health Juba South Sudan; ^9^ Johns Hopkins University Baltimore Maryland USA; ^10^ Division of Tropical and Humanitarian Medicine Geneva University Hospitals Geneva Switzerland; ^11^ Institute of Global Health University of Geneva Geneva Switzerland; ^12^ Center of Vaccinology, Department of Pathology and Immunology University of Geneva Geneva Switzerland

## Abstract

Accurate and accessible diagnostics for hepatitis E virus (HEV) are essential for outbreak preparedness and surveillance, particularly in low‐resource settings. Dried blood spots (DBS) are a scalable alternative to serum, but their diagnostic performance for HEV remains poorly characterised. Paired DBS and serum samples were collected from suspected HEV cases during an outbreak in Bentiu, South Sudan. HEV RNA was detected and quantified by RT‐PCR, and anti‐HEV IgM and IgG were measured by ELISA. DBS performance was compared to serum across assays, and associations between DBS positivity and serum viral load were analysed using logistic regression. Among 100 serum RT‐PCR positive samples, 83 were positive by DBS (sensitivity 83.0%, 95% CI: 74.5–89.1), with reduced sensitivity at low viral loads (Ct > 30). All 48 serum RT‐PCR negative samples were negative by DBS (specificity 100%, 95% CI: 92.6–100). DBS achieved 88.3% sensitivity (95% CI: 77.8–94.2) and 100% specificity (95% CI: 91.2–100) for IgM, while IgG sensitivity and specificity reached 95.0% (95% CI: 86.3–98.6) and 97.5%–100%, respectively. Results were consistent across DBS card types and age groups. DBS provide a practical complementary alternative to serum for HEV surveillance and outbreak investigations, particularly where conventional sampling and cold‐chain transport are challenging. However, slightly reduced sensitivity at low viral loads and IgM detection limits their use as a stand‐alone diagnostic approach for acute case detection Further optimisation of elution methods to increase analyte concentration, along with improved storage conditions, could enhance diagnostic sensitivity.

## 1 Introduction

Hepatitis E virus (HEV) infection is probably the most common cause of acute viral hepatitis and jaundice worldwide [1, 2]. It encompasses a single serotype and four human pathogenic genotypes. The prevalence of HEV infection varies between regions and depends on the genotype present. Genotypes 1 and 2 exclusively infect humans and are often associated with large‐scale outbreaks and epidemics in low‐ and middle‐income countries with low drinking water quality. The 2005 Global Burden of Hepatitis E study estimates 20.1 million annual infections, with 3.4 million clinical cases, 70,000 deaths, and 3,000 stillbirths attributed to HEV genotypes 1 and 2 [3]. The true burden of HEV, however, remains unknown, mainly due to the absence of widely available diagnostics and the low specificity of suspected case definitions [4, 5]. Although the overall case fatality rate for HEV infection is around 1%, mortality can reach up to 25% among pregnant women in their third trimester [6, 7]. Studies from Bangladesh have reported that 19%–25% of maternal deaths were associated with jaundice during pregnancy and suggested that HEV may be a causative factor, contributing to maternal mortality and adverse fetal outcomes, such as spontaneous abortion, stillbirths, and preterm delivery [8, 9]. In one study, 26.9% of HEV‐positive pregnant women died, with fulminant hepatic failure identified as the primary cause of death [10]. Together, these findings underscore the urgent need for improved diagnostics and surveillance to reduce the burden of HEV, particularly among vulnerable populations.

Current diagnostic approaches for confirming HEV primarily rely on serological assays such as enzyme‐linked immunosorbent assays (ELISA) for IgM and IgG antibody detection or molecular techniques such as reverse transcription polymerase chain reaction (RT‐PCR), typically performed on plasma or serum samples. These methods are essential for identifying acute infections, distinguishing past exposure, and confirming outbreaks. Their deployment in outbreak‐prone regions, however, is often constrained by insufficient laboratory infrastructure, limited technical capacity, and the logistical challenges of collecting, storing, and transporting blood samples under strict cold chain conditions. These limitations highlight the urgent need for more flexible and accessible sampling methods to support both case detection and population‐level surveillance.

Dried blood spots (DBS) represent a promising solution to these challenges. DBS are minimally invasive, can be collected with a simple finger prick, require limited training, and are more stable during storage and transport compared to venous blood, eliminating the need for a cold chain [11]. Their utility has been demonstrated in the surveillance and diagnosis of multiple infectious diseases, including HIV [12, 13], hepatitis B and C [14, 15], and measles [16, 17]. Despite this, their application in HEV diagnostics remains underexplored. Most available studies on DBS and HEV focus on IgG antibody detection, with generally good concordance reported between DBS and serum samples. A validation study conducted in Bangladesh reported a sensitivity of 81% and a specificity of 97% for DBS‐based IgG assays compared with serum [18]. Similarly, a recent evaluation confirmed that DBS can reliably detect anti‐HEV IgG, reinforcing their potential use in large‐scale seroepidemiological investigations [19]. Additional data from a study in pregnant women in Burkina Faso reported even higher performance, with DBS detecting anti‐HEV IgG at 96.7% sensitivity and 100% specificity compared to serum [20]. In contrast, evidence for IgM detection is more limited and less consistent. While some studies have shown promising accuracy with sensitivities of 86‐91% and specificities up to 100% under controlled conditions [21], others have reported lower performance, including one study that found sensitivity and specificity of 76.7% and 93.8%, respectively, for DBS‐based IgM detection [20]. Similarly, DBS‐based RNA detection by RT‐PCR has shown good correlation with serum, but sensitivity is reduced in cases with low viral loads [22].

Improving HEV diagnostics is important for broader public health goals, including outbreak preparedness, transmission prevention, surveillance, and vaccine evaluation. Expanding the use of DBS could offer a practical and scalable solution for HEV diagnostics in low‐resource settings. If DBS can be reliably used to identify both recent and past HEV infections, this would greatly expand surveillance capacity and simplify logistics for operational research and outbreak investigations.

To address these gaps, we investigated the performance of DBS compared to serum for both molecular and serological HEV diagnostics. We took advantage of paired serum and DBS samples collected from suspected hepatitis E cases during an outbreak in Bentiu, South Sudan, to understand the trade‐offs in test performance. Specifically, we assessed detection of HEV RNA by RT‐PCR and anti‐HEV IgM and IgG antibodies by ELISA. By combining molecular and serological assessments, this study provides key evidence on the utility of DBS for HEV diagnostics in outbreak‐prone, resource‐limited settings.

## 2 Methods

### 2.1 Study Setting

An enhanced hepatitis E surveillance system was set up to support operational research during the first emergency deployment of hepatitis E vaccine in the Bentiu Internally Displaced Person camp with approvals from the Médecins Sans Frontières (MSF) Ethics Review Board (ERB #2167) and by the South Sudan Ministry of Health Research Ethics Board (RERB‐MOH #54/27/09/2022) [23]. All individuals presenting to the Médecins Sans Frontières (MSF) hospital with suspected hepatitis E, defined as acute jaundice syndrome (AJS), characterised by the sudden or recent onset of yellow discoloration of the eyes or skin, often accompanied by dark urine and/or pale clay stools [24], were identified by clinicians and referred to the study team following their consultation or hospital admission. Study staff explained the objectives of the study to each suspected case. For those willing to participate, informed consent procedures were conducted: adults provided written informed consent, while individuals under 18 years of age provided written assent in conjunction with written consent from a parent or legal guardian. Upon consent, study staff administered a standardised questionnaire to collect information on symptoms, date of symptom onset, vaccination status, and sociodemographic details, including the participant's address within the camp.

### 2.2 Overview of Specimen Collection, Storage and Processing

A laboratory technician collected both venous blood and DBS and prepared all specimens for storage and transport. Venous blood was drawn, after which serum was separated by centrifugation at the MSF hospital. The samples were initially stored at the hospital in Bentiu and transported to Juba under −20°C conditions. Storage in Juba and shipment to Geneva was conducted at −80°C. For DBS preparation, drops of venous blood were spotted directly onto standardised cellulose‐based filter paper cards designed for biological sample collection. Two types of cards were used: Whatman 903 Protein Saver Cards (*n* = 40, Cytiva, Marlborough, MA, USA, Cat. No.10531018) and Ahlstrom‐Munksjö Biosample Collection Cards (*n* = 131, Ahlstrom‐Munksjö, Helsinki, Finland, Cat. No. 8.460.0012.A). Whatman 903 cards are composed of 100% pure cotton fibre and are widely used for clinical DBS applications, including newborn screening, due to their standardised thickness, high absorbency, and validated performance in diagnostic assays [25]. Ahlstrom‐Munksjö Biosample Collection Cards are similarly manufactured from high‐purity cellulose specifically for biological sample collection and in‐vitro diagnostic applications, ensuring consistent blood absorption and analyte stability. After spotting, DBS cards were dried at room temperature. Once completely dried, each DBS card was individually placed in small plastic bags with a single desiccant to limit humidity exposure. All samples were then transported from the field to the Geneva Centre for Emerging Viral Diseases at the Geneva University Hospitals, Switzerland. Serum samples were shipped on dry ice with temperature loggers to ensure a −80°C cold chain during transport, while DBS were transported at room temperature. Upon arrival in Geneva, serum samples were stored at −80°C until testing, while DBS were kept at ambient temperature under dry conditions until analysis.

### 2.3 Sample Selection

**Figure 1:**
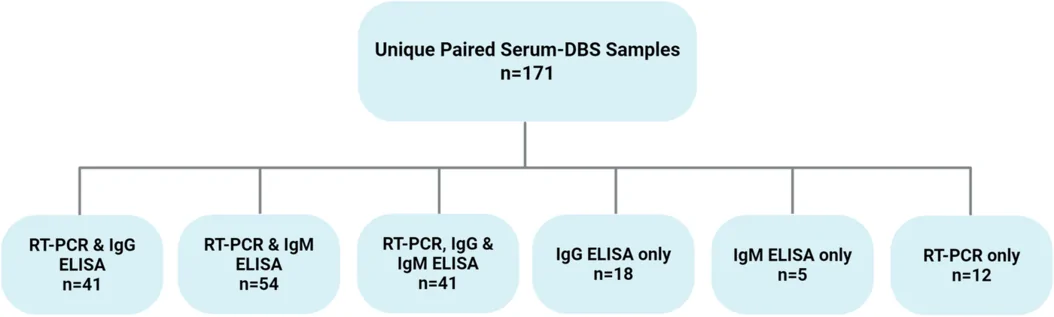
Distribution of paired serum and DBS samples for molecular and/or serological analyses. A total of 171 unique paired serum and DBS samples from suspected HEV cases were included. Samples were grouped according to the combination of molecular and serological analyses performed: 41 samples were evaluated by RT‐PCR and IgG ELISA, 54 by RT‐PCR and IgM ELISA, and 41 by RT‐PCR, IgG ELISA, and IgM ELISA. An additional 18 samples were evaluated by IgG ELISA only, 5 by IgM ELISA only, and 12 by RT‐PCR only.

A total of 171 unique paired serum and DBS samples collected from suspected HEV cases during an outbreak were included in the study. Samples were selected for molecular and/or serological analyses according to sample availability. Of these, 41 samples were tested by both RT‐PCR and IgG ELISA, 54 by both RT‐PCR and IgM ELISA, and 41 by all three assays (RT‐PCR, IgG ELISA, and IgM ELISA). In addition, 18 samples were tested exclusively by IgG ELISA, 5 exclusively by IgM ELISA, and 12 exclusively by RT‐PCR (Figure 1).

For the evaluation of RT‐PCR, 148 paired DBS and serum samples were selected, including 100 serum‐positive and 48 serum‐negative samples. All samples included in this study, both RT‐PCR‐positive and RT‐PCR‐negative were collected within the same outbreak investigation setting (Figure 1). To ensure representation across a range of viral loads among the samples, serum samples were stratified based on their cycle threshold (Ct) values into four groups: < 25, 25–30, 30–45 and > 45 (i.e. the negative samples). The study design aimed to include approximately one‐fourth of the samples from each Ct range. However, due to the limited number of samples with Ct values < 25, only 20 were available for inclusion in this group. Despite this slight imbalance, the final distribution was considered sufficient for robust comparison of DBS and serum performance.

For ELISA testing, 100 paired DBS and serum samples were selected, consisting of 60 ELISA‐positive and 40 ELISA‐negative serum samples. The study design was intentionally enriched for positive samples to enable robust estimation of diagnostic sensitivity while maintaining an adequate number of negative samples for specificity analysis. This enriched distribution does not reflect real‐world disease prevalence and was not intended for direct estimation of predictive values. Selection was carried out in two steps: first, individuals who had already been tested by RT‐PCR, ensuring that diagnostic results were available. As most of these individuals were seropositive for anti‐HEV IgM and IgG, they provided the majority of positive cases. Additional negative samples were then included to reach the target of 100 pairs.

### 2.4 Dried Blood Spots (DBS) Preparation

For RNA extraction and RT‐PCR, two spots from the DBS cards were punched using a hole puncher and placed into an Eppendorf tube containing 1 mL of EasyMAG lysis buffer (BioMérieux, Marcy‐l'Étoile, France). Samples were incubated at room temperature for 30 min with slow vortexing to facilitate release of nucleic acids from the paper matrix. Following incubation, the lysates were centrifuged to remove residual filter paper debris. Based on spot input volumes (~50–80 µL) and the typical recovery efficiency of ~40%–90%, each spot corresponds to approximately 20–70 µL of whole blood, equivalent to about 10–40 µL of serum [26]. The clarified supernatant was then extracted and the RNA was quantified by RT‐PCR. For comparison, RNA extraction from serum samples was performed using 200 µL of input material.

For ELISA, one spot from each DBS card was punched and placed in an Eppendorf tube containing 500 µL of elution buffer consisting of phosphate‐buffered saline (Gibco PBS, Thermo Fisher Scientific) supplemented with 0.05% Tween‐20 (PanReac AppliChem ITW Reagents) and 0.08% sodium azide (NaN_3_, Sigma‐Aldrich) [27]. Samples were incubated at room temperature on a laboratory shaker for 4 h to ensure effective antibody extraction. Following incubation, eluates were centrifuged to remove paper debris, and the supernatants were used for ELISA. Considering that each DBS spot contained approximately 50–80 µL of whole blood and that antibody recovery efficiency typically ranges from 40% to 90%, this corresponds to about 20–70 µL of whole blood, equivalent to approximately 10–40 µL of serum after elution [26]. For ELISA testing, 10 µL of the prediluted eluate was used per assay, while serum ELISAs were performed with 10 µL of serum.

### 2.5 RNA Extraction, Reverse Transcription Polymerase Chain Reaction (RT‐PCR) and Viral Load Quantification

RNA was extracted from DBS samples using the NucliSens easyMAG instrument (BioMérieux, Marcy‐l'Étoile, France), according to the manufacturer's instructions. HEV RNA detection and quantification were performed using the ampliCube HEV 2.0 Quant real‐time quantitative PCR (Mikrogen diagnostik, Neuried, Germany), which is designed for the detection and quantification of genomic RNA of HEV genotypes 1, 2, 3, and 4. The assay was carried out according to the manufacturer's protocol. Viral load quantification was achieved by interpolating Ct values against a standard curve generated from quantified HEV RNA calibrators included in the kit. Results were expressed as international units per millilitre (IU/mL), taking into account the sample input and elution volumes: 200 µL for serum and 45 µL for DBS, with an elution volume of 50 µL for both. Samples were considered positive when the viral load was > 50 IU/mL. Amplification was performed on a CFX96 Thermal Cycler (Bio‐Rad Laboratories, Hercules, USA), allowing simultaneous detection of HEV‐specific RNA and the internal control within a single reaction.

### 2.6 Enzyme‐Linked Immunosorbent Assay (ELISA)

The Wantai HEV‐IgM ELISA (WE‐7196) and Wantai HEV‐IgG ELISA (WE‐7296) kits (Wantai BioPharm, Beijing, China) are commercial assays for the qualitative detection of anti‐HEV antibodies. They use a two‐step incubation, solid‐phase capture ELISA in which recombinant HEV antigens coated on microwells bind specific IgM or IgG antibodies from the sample. Bound antibodies are detected with an enzyme‐conjugated secondary antibody and visualised by a colorimetric reaction. For each sample, the absorbance value (S) is divided by the cut‐off value (CO) to obtain the S/CO ratio. The cut‐off value is calculated for each plate based on the mean absorbance of the negative controls and according to the manufacturer, S/CO ≥ 1.1 is positive, 0.9–1.1 borderline, and < 0.9 negative. Each kit includes positive and negative controls, and all reagents required for the assay.

### 2.7 Statistical Analyses

Sensitivity and specificity were calculated with corresponding 95% binomial confidence intervals (CIs) using the Wilson/Brown hybrid method as implemented in GraphPad Prism. To assess factors associated with DBS positivity, a multivariable logistic regression model was fitted in R (version 4.4). Explanatory variables added as linear terms included serum viral load, DBS card type (Whatman 903 or Ahlstrom‐Munksjö), storage duration and participant age group (< 5, 5–14, and > 15 years). All other analyses, as well as graphical visualisations, were performed in GraphPad Prism and Excel.

## 3 Results

We selected 171 unique blood samples from suspected hepatitis E cases to compare diagnostic performance on DBS versus serum across three assays: RT‐PCR, IgM ELISA and IgG ELISA (Table 1). The study population included 58 children under 5 years, 52 aged 5–14 years and 61 individuals over 15 years, with 74 (43.3%) females. The interval between sample collection and DBS testing ranged from 106 to 154 weeks.

**Table 1:** Description of the study population for RT‐PCR and ELISA assays. Numbers represent paired DBS and serum samples collected from suspected HEV cases from Bentiu Internally Displaced Person camp, 2022. For RT‐PCR, Ct categories were defined based on serum results.

Assay	Total samples	Female (*n*)	Male (*n*)	Positives (*n*)	Negatives (*n*)	Age groups (*n*)	RT‐PCR Ct categories (*n*)
RT‐PCR (Mikrogen diagnostik)	148	70	78	100	48	50 (< 5 years), 45 (5–14 years), 53 (> 15 years)	20 (Ct < 25), 43 (Ct 25–30), 37 (Ct 30–45), 48 (Ct > 45)
IgM ELISA WE‐7196 (Wantai BioPharm)	100	47	53	60	40	23 (< 5 years), 28 (5–14 years), 49 (> 15 years)	—
IgG ELISA WE‐7296 (Wantai BioPharm)	100	43	57	60	40	46 (< 5 years), 33 (5–14 years), 21 (> 15 years)	—

### 3.1 RNA Detection With PCR

**Figure 2:**
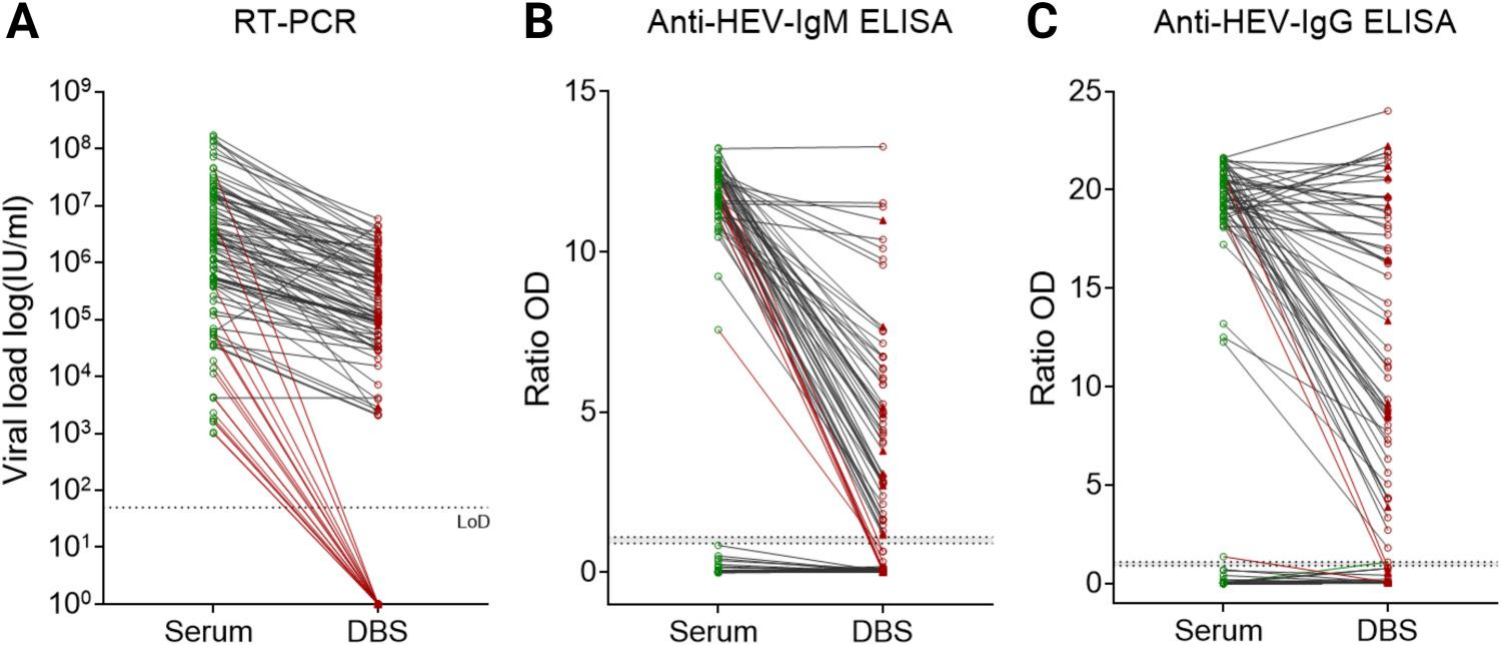
Comparison of DBS and paired serum samples for HEV. Results are shown for samples collected from suspected HEV cases during the Bentiu outbreak by (A) RT‐PCR, (B) anti‐HEV‐IgM ELISA, and (C) anti‐HEV‐IgG ELISA. Serum results are shown in green and DBS results in red. The red line indicates samples that were positive in serum but turned negative in DBS assays, while the green line marks the serum‐negative sample that was classified as borderline/indeterminate in the DBS assay. DBS samples collected on Ahlstrom‐Munksjö cards are represented by circles, whereas those collected on Whatman 903 cards are shown as triangles.

For RT‐PCR analyses, we selected DBS samples from 100 patients with serum samples confirmed positive by RT‐PCR and obtained paired DBS specimens to capture a broad range of viral loads, as well as from 48 patients whose serum samples were confirmed negative by RT‐PCR. The study population comprised 70 females and 78 males, including 50 children under 5 years, 45 aged 5–14 years, and 53 individuals older than 15 years. To assess performance across different levels of viremia, serum samples were stratified according to their Ct values into four groups: < 25 (*n* = 20), 25–30 (*n* = 43), 30–45 (*n* = 37) and > 45 (*n* = 48). Of the 100 serum samples positive by RT‐PCR, 83 were also positive using paired DBS samples, corresponding to a sensitivity of 83.0% (95% CI: 74.5–89.1) (Table 2). Ct values obtained from DBS showed strong agreement with those from serum (Spearman ρ = 0.74), indicating good correlation between specimen types. Of the 17 DBS‐negative samples, 15 had serum Ct values > 30, suggesting reduced sensitivity of DBS at lower viral loads (Figure 2A). Multivariable logistic regression analysis indicated that for every one‐log increase in serum viral load, the odds of DBS positivity increased approximately twofold (Odds Ratio 2.1, 95% CI 1.54–3.10), with no significant differences observed by DBS card type, storage duration or age group (Supplementary Table S1). All 48 paired DBS samples from serum RT‐PCR‐negative patients tested negative (not shown), resulting in a specificity of 100% (95% CI: 92.6–100) (Table 2).

**Table 2:** Diagnostic performance of DBS testing. Sensitivity and specificity for detection of HEV RNA, IgM, and IgG in DBS using serum as the reference standard.

Biomarker	True positives/total positives	Sensitivity (%)	True negatives/total negatives	Specificity (%)
RT‐PCR (Mikrogen diagnostik)	83/100	83.0 (95% CI: 74.5–89.1)	48/48	100 (95% CI: 92.6–100)
IgM ELISA WE‐7196 (Wantai BioPharm)	53/60	88.3 (95% CI: 77.8–94.2)	40/40	100 (95% CI: 91.2–100)
IgG ELISA WE‐7296 (Wantai BioPharm)	57/60	95.0 (95% CI: 86.3–98.6)	39/40	97.5 (95% CI: 87.1–99.9)

### 3.2 Antibodies Detection With ELISA

For ELISA testing, a set of 100 paired DBS and serum samples was selected, with 60 IgM‐positive and 40 IgM‐negative serum samples to allow a more robust evaluation of diagnostic sensitivity and specificity of DBS compared to serum. Among these, IgM analyses included 47 females and 53 males, distributed across three age groups: 23 children under 5 years, 28 aged 5–14 years and 49 individuals older than 15 years. For IgG analyses, 43 females and 57 males were included, with the following age distribution: 46 under 5 years, 33 aged 5–14 years and 21 over 15 years.

Of the 60 DBS samples corresponding to serum IgM‐positive results by ELISA, 53 were also positive by DBS (sensitivity = 88.3%, 95% CI: 77.8–94.2) and all 40 serum IgM‐negatives samples were correctly identified as negative by DBS, resulting in a specificity of 100% (95% CI: 91.2–100) (Figure 2B, Table 2). Among the anti‐HEV IgM‐negative samples, six were RT‐PCR positive, four of which had Ct values > 30. For IgG, DBS correctly identified 57 of 60 serum IgG‐positive samples, corresponding to a sensitivity of 95.0% (95% CI: 86.3–98.6). Among the 40 serum IgG‐negative samples, 39 were negative and one was borderline/indeterminate, yielding a specificity of 97.5% (95% CI: 87.1–99.9) if the borderline is considered positive, or 100% (95% CI: 91.2–100) if considered negative (Figure 2C, Table 2). Similarly, among the anti‐HEV IgG‐negative samples, two were RT‐PCR positive, including one with a Ct value > 30.

## 4 Discussion

This study evaluated the performance of DBS for the detection of HEV RNA and anti‐HEV antibodies using paired serum and DBS samples collected from suspected HEV cases during an outbreak in Bentiu, South Sudan. By assessing both molecular and serological assays within the same outbreak cohort, this study provides a comprehensive evaluation of DBS as a diagnostic approach for HEV in a resource‐limited setting. Using paired serum and DBS samples, which were stored for up to 35 months after collection, we observed that DBS‐based RT‐PCR detected HEV RNA in 83% of serum‐positive cases, with strong correlation between Ct values from DBS and serum (Spearman ρ = 0.74). Discordant results for RT‐PCR were primarily observed in samples with low viral loads (Ct > 30), reflecting a reduction in sensitivity at the lower limit of detection. These findings are consistent with earlier work during a HEV outbreak in Darfur, where DBS and serum RT‐PCR results showed 90.6% concordance [22]. Importantly, all 48 serum‐negative samples were also negative by DBS, corresponding to a specificity of 100% (95% CI: 92.6–100). In outbreak settings, where rapid case detection is essential for transmission control, diagnostic sensitivity becomes particularly critical, as false‐negative results may contribute to underestimation of active infection and delayed public health response. Therefore, the reduced sensitivity observed for DBS RT‐PCR in HEV, particularly among individuals with low viral loads or early‐stage infection, should be considered when interpreting its operational utility for acute case detection. Although a sensitivity of 83% may be insufficient for primary case detection during outbreak control, DBS RT‐PCR offers a practical alternative in settings where conventional sampling and cold‐chain transport are challenging, with negative results interpreted with caution when clinical suspicion remains high.

For serological testing, our findings confirm that DBS‐based ELISA can reliably detect HEV antibodies, though performance varies by antibody class. For IgG, DBS detected 57 of 60 serum‐positive samples and 39 of 40 serum‐negative samples, confirming excellent agreement for detecting past HEV exposure, with 95.0% sensitivity and 97.5% specificity. This accuracy is in line with prior studies, which reported sensitivities of 81%–96.7% and specificities of 97%–100% for DBS IgG [18, 20]. For recent or acute HEV infection, IgM detection from DBS identified 53 of 60 serum‐positive samples and all 40 serum‐negative samples (sensitivity 88.3%, specificity 100%), indicating slightly reduced sensitivity compared to serum but very high specificity. These results are consistent with previous reports [21]. This reduction may reflect lower antibody concentrations in some samples, though we did not conduct quantitative ELISAs in this study. In outbreak settings, where IgM detection can support the identification of recent infections and ongoing transmission, a sensitivity of 88.3%, compared to using a blood sample directly, may limit the use of DBS IgM as a stand‐alone diagnostic tool for early case confirmation. However, the high specificity observed indicates that DBS IgM can provide valuable complementary information for outbreak investigations, particularly in settings where serum collection and cold‐chain transport are challenging. Negative DBS IgM results should therefore be interpreted cautiously in individuals with a strong clinical or epidemiological suspicion of acute HEV infection and may require confirmation using serum‐based testing when feasible. Notably, a small number of IgG‐ or IgM‐negative samples were positive for HEV RNA, most with high Ct values (> 30), suggesting low‐level viremia or early infection prior to seroconversion. These findings highlight the complementary value of molecular testing in detecting early HEV infections before seroconversion of patients. Overall, DBS are well suited for large‐scale IgG seroprevalence studies and can be used for IgM detection, with some sensitivity challenges if stored in similar conditions.

This study has several important limitations. The first relates to differences in sample volume between DBS and serum, which likely contributed to reduced sensitivity, particularly for RT‐PCR and IgM detection. For RT‐PCR, RNA was extracted from approximately 45 µL of serum derived from two DBS spots, whereas serum testing used 200 µL of input material. Similarly, for antibody detection, only one DBS spot (corresponding to ~25 µL of serum diluted in buffer, of which 10 µL was applied to the assay) was used, compared with 10 µL of serum used in ELISA. These discrepancies in input volume and sample matrix could explain lower performance of DBS, particularly in specimens with low viral load or low antibody titres. Sensitivity could potentially be improved by optimising elution protocols, such as using smaller elution volumes to increase analyte concentration. Second, DBS samples in this study were prepared from venous blood rather than true capillary collection, which may limit comparability with field conditions where finger‐prick sampling is typically used. Previous research in other infectious disease systems, including SARS‐CoV‐2 [28], HIV [29], and dengue [30, 31], have generally shown that antibody and viral measurements obtained from capillary blood closely align with those from venous samples. However, minor discrepancies, particularly for IgM detection or at low viral concentrations have been reported. Third, DBS and serum samples were not tested simultaneously, and the storage duration and conditions for DBS were not optimal, raising the possibility that RNA degradation or antibody instability may have led to lower apparent sensitivity of DBS. Different storage conditions, such as maintaining DBS cards at 4°C instead of room temperature, and storage durations could help preserve RNA and antibody integrity over time resulting in increased sensitivity. Concurrent testing of paired specimens would also minimise these effects and enable more accurate assessment of DBS performance without the effect of storage or time. Although no significant differences were observed between the two DBS card types used in this study, subtle variations related to haematocrit effects, blood absorption properties, or analyte stability between different filter paper substrates cannot be completely excluded. Comparative studies evaluating similar Ahlstrom cellulose‐based filter papers, against Whatman 903 have demonstrated overall good agreement for HIV‐1 viral load classification, although somewhat lower sensitivities were observed [32]. These factors should be considered during assay validation and standardisation. Furthermore, the limited blood volume to DBS prevented precise quantification of viral load or antibody levels, restricting direct comparisons with serum and limiting interpretation of absolute concentrations. Importantly, the ELISA cut‐off values applied in this study were originally validated for serum specimens and may not fully reflect the DBS matrix. Future investigations should explore this aspect, including direct comparisons of ELISA cut‐off values between serum and DBS samples to establish matrix‐appropriate thresholds. Finally, our study was conducted in the context of an outbreak and predominantly included symptomatic individuals. As a result, the study population may not fully represent the broader spectrum of infection observed in community or non‐outbreak settings. Therefore, the diagnostic performance observed here may not be directly generalisable to other populations.

Overall, our findings demonstrate that DBS prepared from venous blood represent a feasible and practical alternative to serum for HEV diagnostics, particularly in field and outbreak settings. A major strength of this study is the comprehensive evaluation of DBS for both molecular detection of HEV RNA and serological detection of anti‐HEV IgM and IgG using paired specimens collected during an outbreak. This provides evidence for the applicability of DBS across multiple stages of HEV infection, from acute viremia to antibody detection. While DBS in this study were prepared from venous blood, the approach has the potential to be even less invasive if applied to capillary blood collected via finger prick. Their collection requires minimal training, and facilitates transport and storage without a strict cold chain, advantages that are especially valuable in resource‐limited environments. By allowing detection of both recent and past infections, DBS can strengthen surveillance systems and facilitate operational research where conventional blood sampling is logistically challenging. While sensitivity is somewhat lower for samples with low viral load, and presumably with low‐titre IgM, performance for IgG detection appears robust and IgM detection remains acceptable, supporting their application in both seroprevalence studies and outbreak investigations.

In conclusion, this study demonstrates that DBS from venous blood provide a reliable and efficient approach for integrated HEV molecular and serological testing during outbreak investigations. By evaluating HEV RNA together with anti‐HEV IgM and IgG in paired serum and DBS samples collected during an outbreak, this work provides evidence supporting the use of DBS for HEV surveillance and outbreak response in resource‐limited settings. Although sensitivity was lower than serum for samples with low viral load and some acute infections, DBS showed high specificity and acceptable overall performance while offering substantial logistical advantages for simple collection, storage, and transport. These findings provide a foundation for future studies to optimise sampling methods, storage conditions, and assay performance.

## Author Contributions

Andrew S. Azman, Isabella Eckerle and Benjamin Meyer conceptualised the study. Catia Alvarez, Pascale Sattonnet‐Roche, Shimul Das conducted laboratory analyses for the study. Catia Alvarez performed data analysis. Robin Nesbitt and Isabella Eckerle contributed to resources. Andrew S. Azman conducted statistical analysis. Catia Alvarez, Andrew S. Azman and Benjamin Meyer wrote the manuscript. Robin Nesbitt, Kinya Vincent Asilaza, Pascale Sattonnet‐Roche, Joseph Wamala, Shimul Das, Melat Haile, Monica Rull, Etienne Gignoux, Manuel Albela, John Rumunu, Isabella Eckerle, Iza Ciglenecki contributed to reviewing and editing manuscript. Andrew S. Azman, Benjamin Meyer and Isabella Eckerle supervised experiments.

## Conflicts of Interest

B.M. has received research funding from Moderna Inc. outside of this project and serves as a consultant for Rocketvax AG. Médecins Sans Frontières provided salary support for V.A., M.H., M.R., M.A., I.C. and A.S.A. and indirect salary support for Epicentre employees R.N. and E.G. The remaining authors declare no conflicts of interest.

## Supporting information


Supporting File

## Data Availability

The data that support the findings of this study are available from the corresponding author upon reasonable request.

## References

[1] J. H. Hoofnagle , K. E. Nelson , and R. H. Purcell , “Hepatitis E,” New England Journal of Medicine 367, no. 13 (2012): 1237–1244. 23013075 10.1056/NEJMra1204512

[2] R. H. Purcell and S. U. Emerson , “Hepatitis E: An Emerging Awareness of an Old Disease,” Journal of Hepatology 48, no. 3 (2008): 494–503. 18192058 10.1016/j.jhep.2007.12.008

[3] D. B. Rein , G. A. Stevens , J. Theaker , J. S. Wittenborn , and S. T. Wiersma , “The Global Burden of Hepatitis E Virus Genotypes 1 and 2 in 2005,” Hepatology 55, no. 4 (2012): 988–997. 22121109 10.1002/hep.25505

[4] M. S. Hakim , W. Wang , W. M. Bramer , et al., “The Global Burden of Hepatitis E Outbreaks: A Systematic Review,” Liver International 37, no. 1 (2017): 19–31. 10.1111/liv.13237 27542764

[5] G. W. Webb and H. R. Dalton , “Hepatitis E: An Underestimated Emerging Threat,” Therapeutic Advances in Infectious Disease 6 (2019): 2049936119837162. 30984394 10.1177/2049936119837162 PMC6448100

[6] A. Bergløv , S. Hallager , and N. Weis , “Hepatitis E during Pregnancy: Maternal and Foetal Case‐Fatality Rates and Adverse Outcomes—A Systematic Review,” Journal of Viral Hepatitis 26, no. 11 (2019): 1240–1248. 31095813 10.1111/jvh.13129

[7] N. Kamar , R. Bendall , F. Legrand‐Abravanel , et al., “Hepatitis E,” Lancet 379, no. 9835 (2012): 2477–2488. 22549046 10.1016/S0140-6736(11)61849-7

[8] E. S. Gurley , A. K. Halder , P. K. Streatfield , et al., “Estimating the Burden of Maternal and Neonatal Deaths Associated With Jaundice in Bangladesh: Possible Role of Hepatitis E Infection,” American Journal of Public Health 102, no. 12 (2012): 2248–2254. 23078501 10.2105/AJPH.2012.300749 PMC3519295

[9] S. Patra , A. Kumar , S. S. Trivedi , M. Puri , and S. K. Sarin , “Maternal and Fetal Outcomes in Pregnant Women With Acute Hepatitis E Virus Infection,” Annals of Internal Medicine 147, no. 1 (2007): 28–33. 17606958 10.7326/0003-4819-147-1-200707030-00005

[10] A. Kumar , M. Beniwal , P. Kar , J. B. Sharma , and N. S. Murthy , “Hepatitis E in Pregnancy,” International Journal of Gynecology & Obstetrics 85, no. 3 (2004): 240–244. 15145258 10.1016/j.ijgo.2003.11.018

[11] M. D. Lim , “Dried Blood Spots for Global Health Diagnostics and Surveillance: Opportunities and Challenges,” American Journal of Tropical Medicine and Hygiene 99, no. 2 (2018): 256–265. 29968557 10.4269/ajtmh.17-0889 PMC6090344

[12] S. S. Solomon , S. Solomon , I. I. Rodriguez , et al., “Dried Blood Spots (DBS): A Valuable Tool for HIV Surveillance in Developing/Tropical Countries,” International Journal of STD & AIDS 13, no. 1 (2002): 25–28. 11802926 10.1258/0956462021924578

[13] A. Johannessen , “Dried Blood Spots in HIV Monitoring: Applications in Resource‐Limited Settings,” Bioanalysis 2, no. 11 (2010): 1893–1908. 21083497 10.4155/bio.10.120

[14] B. Lange , J. Cohn , T. Roberts , et al., “Diagnostic Accuracy of Serological Diagnosis of Hepatitis C and B using Dried Blood Spot Samples (DBS): Two Systematic Reviews and Meta‐Analyses,” supplement, BMC Infectious Diseases 17, no. Suppl 1 (2017): 700. 29143672 10.1186/s12879-017-2777-y PMC5688450

[15] S. Bibi , T. R. Siddiqui , S. E. Alam , W. Ahmed , and H. Qureshi , “Comparison of Dried Blood Spots With Conventional Blood Sampling for Diagnosis of Hepatitis B & C Through Serological and Molecular Technique; a Pilot Study,” JPMA. The Journal of the Pakistan Medical Association 70, no. 7 (2020): 1214–1219. 32799276 10.5455/JPMA.23293

[16] K. E. Colson , A. Potter , C. Conde‐Glez , et al., “Use of a Commercial ELISA for the Detection of Measles‐Specific Immunoglobulin G (IgG) in Dried Blood Spots Collected from Children Living in Low‐Resource Settings: Detection of Measles IgG in DBS,” Journal of Medical Virology 87, no. 9 (2015): 1491–1499. 25988945 10.1002/jmv.24136

[17] A. Uzicanin , I. Lubega , M. Nanuynja , et al., “Dried Blood Spots on Filter Paper as an Alternative Specimen for Measles Diagnostics: Detection of Measles Immunoglobulin M Antibody by a Commercial Enzyme Immunoassay,” supplement, Journal of Infectious Diseases 204, no. suppl_1 (2011): S564–S569. 21666214 10.1093/infdis/jir088

[18] J. Øverbø , A. Aziz , K. Zaman , et al., “Stability and Feasibility of Dried Blood Spots for Hepatitis E Virus Serology in a Rural Setting,” Viruses 14, no. 11 (2022): 2525. 36423134 10.3390/v14112525 PMC9692628

[19] R. Sultana , T. R. Bhuiyan , A. S. Sathi , et al., “Developing and Validating a Modified Enzyme Linked Immunosorbent Assay Method for Detecting HEV IgG Antibody from Dried Blood Spot (DBS) Samples in Endemic Settings,” Microbes and Infection 24, no. 2 (2022): 104890. 34628012 10.1016/j.micinf.2021.104890 PMC8960178

[20] C. Chhoung , S. Ouoba , M. Lingani , et al., “Weighted Prevalence and Associated Risk Factors of Hepatitis E Virus Antibodies Among Pregnant Women in Rural Burkina Faso using Dried Blood Spot Samples,” Hepatology Research 55, no. 6 (2025): 795–806. 40318091 10.1111/hepr.14180

[21] M. P. Singh , M. Majumdar , B. Budhathoki , K. Goyal , Y. Chawla , and R. K. Ratho , “Assessment of Dried Blood Samples as an Alternative Less Invasive Method for Detection of Hepatitis E Virus Marker in an Outbreak Setting,” Journal of Medical Virology 86, no. 4 (2014): 713–719. 24375126 10.1002/jmv.23874

[22] A. Mérens , P. J. Guérin , J. P. Guthmann , and E. Nicand , “Outbreak of Hepatitis E Virus Infection in Darfur, Sudan: Effectiveness of Real‐Time Reverse Transcription‐PCR Analysis of Dried Blood Spots,” Journal of Clinical Microbiology 47, no. 6 (2009): 1931–1933. 19339474 10.1128/JCM.02245-08 PMC2691126

[23] I. Ciglenecki , J. Rumunu , J. F. Wamala , et al., “The First Reactive Vaccination Campaign against Hepatitis E,” Lancet Infectious Diseases 22, no. 8 (2022): 1110–1111. 35870452 10.1016/S1473-3099(22)00421-2

[24] A. Koyuncu , R. Nesbitt , C. Alvarez , et al., “Diagnostic Performance and Kinetics of Hepatitis E Viral RNA and IgM Antibody Test Positivity in a Genotype 1 Outbreak in South Sudan,” Journal of Infectious Diseases 233 (2025): e250–e258, 10.1093/infdis/jiaf436. PMC12811865 40811666

[25] P. W. Smit , K. A. Sollis , S. Fiscus , et al., “Systematic Review of the Use of Dried Blood Spots for Monitoring HIV Viral Load and for Early Infant Diagnosis,” PLoS One 9, no. 3 (2014): e86461. 24603442 10.1371/journal.pone.0086461 PMC3945725

[26] K. R. Baillargeon , J. C. Brooks , P. R. Miljanic , and C. R. Mace , “Patterned Dried Blood Spot Cards for the Improved Sampling of Whole Blood,” ACS Measurement Science Au 2, no. 1 (2022): 31–38. 35211698 10.1021/acsmeasuresciau.1c00031 PMC8855418

[27] N. Grüner , O. Stambouli , and R. S. Ross , “Dried Blood Spots—Preparing and Processing for Use in Immunoassays and in Molecular Techniques,” Journal of Visualized Experiments: JoVE 13, no. 97 (2015): 52619, 10.3791/52619. PMC4397000 25867233

[28] M. Khan , C. Rosadas , K. Katsanovskaja , et al., “Simple, Sensitive, Specific Self‐Sampling Assay Secures SARS‐CoV‐2 Antibody Signals in Sero‐Prevalence and Post‐Vaccine Studies,” Scientific Reports 12, no. 1 (2022): 1885. 35115570 10.1038/s41598-022-05640-x PMC8814240

[29] N. Tang , V. Pahalawatta , A. Frank , et al., “HIV‐1 Viral Load Measurement in Venous Blood and Fingerprick Blood using Abbott RealTime HIV‐1 DBS Assay,” Journal of Clinical Virology 92 (2017): 56–61. 28531553 10.1016/j.jcv.2017.05.002

[30] S. Matheus , J. B. Meynard , V. Lacoste , J. Morvan , and X. Deparis , “Use of Capillary Blood Samples as a New Approach for Diagnosis of Dengue Virus Infection,” Journal of Clinical Microbiology 45, no. 3 (2007): 887–890. 17229857 10.1128/JCM.02063-06 PMC1829093

[31] M. Zahreddine , B. Parra , L. Pierce , et al., “High Correlation between Detection of Dengue IgG from Dried Blood Spots and Serum using an Indirect IgG ELISA Assay: A Validation Study in Fortaleza, Brazil,” PLoS Neglected Tropical Diseases 19, no. 2 (2025): e0012880. 40019871 10.1371/journal.pntd.0012880 PMC11893133

[32] E. Rottinghaus , E. Bile , M. Modukanele , et al., “Comparison of Ahlstrom Grade 226, Munktell TFN, and Whatman 903 Filter Papers for Dried Blood Spot Specimen Collection and Subsequent HIV‐1 Load and Drug Resistance Genotyping Analysis,” Journal of Clinical Microbiology 51, no. 1 (2013): 55–60. 23077127 10.1128/JCM.02002-12 PMC3536251

