# Supplementary material for: Use of Dried Blood Spots for Identifying Hepatitis E Infections in an Outbreak Setting
Source: J Med Virol. 2026 Sep 15;98(9):e71155. doi: 10.1002/jmv.71155 (PMC13576809; doi:10.1002/jmv.71155)
Supplement: Supplementary file 1 [file JMV-98-e71155-s001.docx]

**Table S1. Overview of coefficient estimates from the logistic regression model evaluating PCR stability in DBS samples.**

| Term | OR | 95% CI | p-value |
| --- | --- | --- | --- |
| Log serum viral load (IU/mL) | 2.06 | (1.54, 3.10) | <0.0001 |
| Storage duration (days, centered) | 1.01 | (1.00, 1.02) | <0.20 |
| Age 5-14 vs. 0-4 | 1.13 | (0.16, 8.12) | <0.90 |
| Age 15+ vs. 0-4 | 1.53 | (0.26, 9.73) | <0.64 |
| Whatman 903 vs. reference card | 0.50 | (0.10, 2.70) | <0.41 |
| Outcome: DBS viral load detectable (>50 IU/mL). Odds ratios and 95% CIs from profile likelihood (confint() in R). | | | |
